# A Randomized Control Trial for ReDeSign: A Dementia-Friendly Mobile Microlearning Training for Store Workers in Japan

**DOI:** 10.1093/geront/gnac182

**Published:** 2022-12-12

**Authors:** Hiroshige Matsumoto, Yasuhiro Hagiwara, Noriko Yamamoto-Mitani, Ayumi Igarashi

**Affiliations:** Department of Community Health Nursing, Graduate School of Medicine, The University of Tokyo, Tokyo, Japan; Department of Biostatistics, Graduate School of Medicine, The University of Tokyo, Tokyo, Japan; Department of Gerontological Home Care and Long-term Care Nursing, Graduate School of Medicine, The University of Tokyo, Tokyo, Japan; Department of Gerontological Home Care and Long-term Care Nursing, Graduate School of Medicine, The University of Tokyo, Tokyo, Japan

**Keywords:** Dementia-friendly training, Helping behavior, Mobile microlearning, Social engagement

## Abstract

**Background and Objectives:**

Dementia-friendly training should be incorporated in neighborhood stores for people living with dementia to maintain engagement in social activities. However, there is a lack of evidence of dementia-friendly training in these workplaces, and existing trainings have time constraints. We developed a mobile microlearning program based on stigma theory and the bystander intervention model. This study aimed to evaluate the microlearning program’s effectiveness.

**Research Design and Methods:**

Convenience store workers in Tokyo were recruited for a randomized, waiting-list, and controlled trial. The intervention group completed a 50-min online course. The primary outcome was an attitude toward people living with dementia. The secondary outcomes were knowledge of dementia and helping behavior toward customers suspected of having dementia. Data were collected at baseline, after 1 month, and 4 months following the randomization.

**Results:**

Process evaluations confirmed satisfaction and high completion rates of the program. In total, 150 participants were included in the analysis. The intervention group showed significantly greater improvements in attitude (Hedge’s *g* = 0.70) and knowledge (*g* = 0.59) after 1 month, compared to the control group. Helping behavior increased in the intervention group, although it did not differ significantly between the groups. All outcomes remained significantly improved after 4 months.

**Discussion and Implications:**

The findings provide evidence that dementia-friendly training reduces the general public’s stigma and increases helping behavior in stores. Mitigation of time constraints through mobile microlearning is expected to contribute to the dissemination and help people living with dementia maintain their social participation in the communities.

**Clinical Trials Registration Number:** UMIN000043623

Social participation of people living with dementia should be ensured not only for their rights (World Dementia Council, 2020) but also to maintain and improve their quality of life (Everard et al., 2000; Heward et al., 2017; Livingston et al., 2017). Because a cure for dementia has not yet been discovered, it is a public health priority to have social and environmental initiatives to foster inclusive communities where people living with dementia are able to participate in society (Alzheimer’s Disease International, 2016; Gan et al., 2021; Lin & Lewis, 2015; Shannon et al., 2019; World Health Organization, 2017). The objective of these dementia-friendly initiatives is to raise people’s awareness of dementia (Hebert & Scales, 2019; Maki et al., 2020; Wiersma & Denton, 2016). Awareness raising campaigns targeting the general public and dementia-friendly training targeting people working in general occupations such as stores, banks, and transportation are widespread (Matsumoto et al., 2021; Shanley et al., 2004).

Dementia-friendly training for store workers is one of the most important programs for encouraging people living with dementia to engage in social and economic activities. Shopping is the most common and preferred reason for them to go out (Bould & Hill, 2018; Duggan et al., 2008). However, they often have difficulty finding and paying for items in the store and may need help from store clerks (Brorsson et al., 2013; Innovations in Dementia, 2011). Difficulties in social participation caused by cognitive decline may lead to maladaptation if appropriate assistance is not provided (Maki et al., 2020). People living with dementia and their families want store workers to be trained about dementia and to be helpful and responsive in emergencies (Smith et al., 2016; Wu et al., 2019). Thus, dementia-friendly initiatives include the establishment of dementia-friendly stores through dementia training for store workers (Cabinet Meeting for Dementia Policy, 2019; Maki et al., 2020; Shannon et al., 2019).

However, there is a lack of evidence for dementia-friendly training for the public, including workers in general occupations. Although existing qualitative and single-group pre–post studies have reported that dementia-friendly training improves knowledge of dementia or attitudes toward dementia in the general public (Matsumoto et al., 2021), there is only one randomized controlled trial (RCT) evaluating dementia-friendly training for the general public (Kim et al., 2021), which reported an absence of significant intervention effects. However, as noted by Kim et al., their study had major limitations of high loss due to a low follow-up rate, and those having high levels of stigma were more likely to not follow-up. This volunteer bias of the participants included in the analysis is considered to have led to an underestimation of the intervention effect.

In addition, it has been repeatedly stated that it is difficult to disseminate dementia-friendly training in general occupations (Dean et al., 2015; Heward et al., 2017; Shannon et al., 2019). For example, the most widespread training program in Japan, the Dementia Supporter Training Course, takes 90 min and is a face-to-face group training program, thus, it has time constraints. Many retail employees are part-time shift workers (Statistics Bureau, 2017), making it more difficult for them to participate in group training.

To address these challenges, a new dementia-friendly training program called ReDeSign (Relational Design for Dementia and Task Significance) was developed and evaluated in an RCT. This study reports the main results of this trial.

ReDeSign adopted mobile microlearning (Bruck et al., 2012) to overcome time constraints. Mobile microlearning, a kind of e-learning, allows participants to take courses on their smartphones, reducing waiting and traveling times for learning. Furthermore, as the content is divided into independent blocks, participants can take the course in fragments even if they do not have continuous free time. It has been shown to motivate participants and to be superior for memory retention (Göschlberger & Bruck, 2017; Jahnke et al., 2020). Mobile microlearning is gradually being adopted by in-house human resource training programs (Leong et al., 2020). The advantages of online interventions include resilience to pandemics such as coronavirus disease 2019 (COVID-19).

## Theoretical Background

The ReDeSign model was designed based on the theory of stigma surrounding mental illness and the bystander intervention model. Stigma about mental illness is a negative and incorrect attitude toward the target population (people living with dementia in this study), yet dementia is plagued by it (Blay & Peluso, 2010; Herrmann et al., 2018). Stigma is not only the psychological concept of stereotype (cognitive aspect) and prejudice (emotional aspect) but is also expressed as discrimination (behavioral aspect; Corrigan & Penn, 1999). The present study considered that the suppression of helping behavior is a behavioral aspect of stigma, and reducing stigma will promote helping behavior. A dual-process model (Pryor et al., 2004) consisting of reflective and deliberative processes, has been proposed as a mechanism by which stigma suppresses helping behavior.

Approaches to reducing stigma are categorized as protest, education, and contact (Corrigan & Penn, 1999), of which education and contact have been shown to be effective (Corrigan et al., 2012). A combination of the two is expected to be more effective (Griffiths et al., 2014; Kim et al., 2021). As face-to-face interaction is difficult to implement on a large scale (Clement et al., 2012), ReDeSign adopted contact through videos, which have been used in the field of psychiatric education (Janoušková et al., 2017). Systematic reviews have concluded that video contact, although inferior to face-to-face contact, is significantly effective (Corrigan et al., 2012), more so than education-only interventions (Janoušková et al., 2017; Yamaguchi et al., 2013). Furthermore, the imagined contact hypothesis proposed by Crisp and Turner (2009) states that mental simulation of social interaction with the target group is as effective as face-to-face contact in reducing stigma and has been reported to be moderately effective (Miles & Crisp, 2014). Based on the imagined contact hypothesis, we created ReDeSign to include simulation games with customers living with dementia, where the consequences of the simulation were more successful interactions with customers.

Previous studies have identified a positive association between knowledge of dementia and helping behavior intentions (Lane & Yu, 2020; Takao & Maki, 2019). The bystander intervention model can explain this association (Matsumoto, Igarashi, Sakka et al., 2022). The bystander intervention model was developed from a series of studies by Latane and Darley, which modeled the decision-making process of implementing a helping behavior in the following steps (Darley & Latane, 1968; Latane & Nida, 1981): (a) noticing the event, (b) interpreting it as an emergency, (c) feeling personally responsible for dealing with it, (d) possessing the necessary skills and resources to act, and (e) intervening. This model suggests that knowledge about the signs and symptoms of dementia facilitates the interpretation of the situation as being caused by dementia (second step) and knowledge about the necessary skills for interacting with a person living with dementia (fourth step). It was also demonstrated that interpretation mediates the association between knowledge of dementia and helping behavior (Matsumoto, Igarashi, Sakka et al., 2022). ReDeSign’s educational content focuses on these aspects.

## Method

ReDeSign-RCT was registered in the UMIN Clinical Trials Registry (UMIN000043623) and approved by the Ethics Committee of the School of Medicine The University of Tokyo (2020348NI). A detailed study protocol and intervention program can be found elsewhere (Matsumoto, Igarashi, Hagiwara et al., 2022).

### Design and Participants

The ReDeSign-RCT was an open-label, randomized, waiting-list, and controlled design. Convenience store workers were identified as participants because people living with dementia rely on small neighborhood stores within walking distance to avoid complex transportation and getting lost (Brorsson et al., 2013). Stores located in Tokyo and franchisees of seven major convenience store franchisers were recruited between May and July 2021. Stores in hospitals or universities were excluded because of their different customer mixes. The participants had to be working at a convenience store, be at least 16 years old, have access to the Internet via a smartphone or PC, and be able to understand Japanese. The employment status of the participants included full-time employees, part-time employees, franchise owners, and family members of the owners who worked at the store. Individual employee participation was not disclosed to the store managers and owners. Informed consent was electronically obtained from the participants.

### Procedure

First, we sent direct mail to store managers based on the franchisers’ official websites. The store managers sent back the number of expected participants through a web form or postcard (store registration). After confirming the registration, the researchers delivered the enrollment cards to the stores.

The participants individually read the study electronically and registered their names and e-mail addresses (individual registration). When individual registration was completed, blocked allocation at a 1:1 ratio was performed successively using a web-based randomization system (Sealed Envelope Ltd, 2021). The individuals were informed of their group allocation following a baseline survey (T1).

The intervention group’s participants were asked to complete the ReDeSign course within 1 month after the T1 survey and to respond to a response survey at the end of the course. The postintervention survey (T2) and a follow-up survey (T3) were conducted 1 month and 4 months after the T1 survey, respectively. Even if they had not completed the ReDeSign course, they responded to the T2 and T3 surveys.

The control group participants completed the T2 survey after a 1-month waiting period. They were then able to use the ReDeSign courses. The length of the waiting period was determined to minimize loss to follow-up.

The participants received rewards worth 500 yen for each survey (T1, T2, and T3), and 1,500 yen for completing the ReDeSign course. The reward amount was equated to the stores’ hourly wages so that the participants were not biased toward those with low levels of stigma. In other words, we prevented the participants who were not interested in dementia (who presumably have a high level of stigma) from dropping out of the study.

### Intervention

The intervention, ReDeSign, was a mobile microlearning program designed for convenience store workers. It consisted of 40 units of microlearning contents arranged in seven lessons, and took 50 min to complete. The main content included (a) simulation games, (b) brief lectures, (c) quizzes, and (d) documentary videos. The course was delivered on a subscription-type learning management system, TalentLMS (Epignosis, 2021), which allowed participants to take the course through smartphones or PCs. A demo version is available on our website (https://nimpro.info/elearning/courses/demo).

### Simulation Games

Four simulation games were developed with each game corresponding to the following early symptoms of dementia: (a) memory and language impairment, (b) disorientation, (c) acalculia, and (d) impaired attention. All the simulation scenarios in this study began with a scene in which a customer who might have dementia enters a convenience store. For example, a customer takes a long time to pay on the counter, and participants are required to choose one of the two response options. If the participant chose the better option (e.g., helping to count coins), they were given positive feedback, after which the next scene was presented. If they choose another option (e.g., rushing to pay), the reason for choosing the better option is explained. These simulations, where the participants experience successful communication with a person living with dementia, are expected to reduce stigma according to the imagined contact hypothesis.

### Lectures

ReDeSign’s lectures were casual and friendly, involving animated videos. The contents were based on the Dementia Supporter Training Course (National Caravan-Mate Coordinating Committee, 2014). The lecture further explained that continuing shopping is key to maintaining a sense of independence and belonging to a community for people living with dementia (Bould & Hill, 2018). Participants were informed that their work in retail stores has a positive impact on customers living with dementia.

### Quizzes

At the end of each lesson, three quizzes were presented, corresponding to the lesson content for memory retention. Immediate feedback was provided for incorrect answers.

### Documentary Videos

The participants watched three documentary videos of people living with dementia (NHK Public Welfare Organization, 2018), which aimed to combat negative stereotypes of dementia. These videos were intended to remind the participants that people can still be autonomous after having dementia, that they should not compensate for what the person can do on their own, and that community involvement is essential.

### Measurements

All measurements were performed at the individual level using a self-administered web survey. The outcome variables were measured three times (T1, T2, and T3). Attitudes, knowledge, and helping behaviors were measured as outcomes, corresponding to the three aspects of stigma mentioned previously: cognitive, emotional, and behavioral.

The primary outcome was an attitude toward people living with dementia, which was measured using a scale developed by Kim and Kuroda (2011). The scale contains 14 items distributed over four subscales: tolerance (five items), refusal (four items), social distance (three items), and affinity (two items). The example items include “I don’t mind if someone with dementia moves into my neighborhood” and “people living with dementia should participate in more community activities.” The responses were rated using a four-point Likert-type scale ranging from 1 (disagree) to 4 (agree), and seven items were reverse scored. The total score (sum of the 14 items) was used in the analysis. Higher scores indicate a more positive attitude. Cronbach’s alpha for the participants in this study was 0.832.

The secondary outcomes were knowledge of dementia and helping behaviors in convenience stores for customers with dementia. The Japanese-language version of the Dementia Knowledge Assessment Scale (DKAS-J; Annear et al., 2017) was used to measure knowledge of dementia. DKAS-J contains 18 statements (items) about dementia, some being factually correct and others being incorrect. The participants answer by selecting one of the four options: false, probably false, probably true, and true. Although the statements cover diverse information about dementia, including pathology, symptomatology, diagnosis, care, communication, and risk factors, the scale is unidimensional. One point is scored for each correct response (“probably true” or “true” for the correct statement), and higher total scores indicate higher knowledge (range: 0–36). Cronbach’s alpha for the participants was 0.734.

Helping behavior was measured using a new scale developed for this study (see Supplementary Table 1). It contains nine items that describe situations where a person living with dementia may require helping behavior in a store, such as “a customer who has lost his/her way home” and “purchases the same items repeatedly.” The participants were asked whether they had encountered them within the past month, and if so, they responded on how often they had engaged in helping behavior using four-point Likert-type answers (1 = never to 4 = always). The average frequency scores of the situations encountered were used in the analysis as the helping behavior score (range: 1–4). The definition of helping behavior includes not only direct helping behavior but also information sharing with supervisors, coworkers, and administrative agencies.

Additionally, course satisfaction (two items: usefulness and satisfaction), completion rate (%), and time spent until completion (minutes) were measured as indicators of process evaluation.

### Sample Size

We estimated the sample size to detect changes in the primary outcome. Medium effect size (*d* = 0.5) was expected based on a previous study (Igarashi et al., 2020) that used the same scale. Under the assumption of 80% power and two-sided alpha level of 0.05, 124 participants (62 per group) were estimated to be sufficient to detect a change in attitude. Twenty-five percent loss to follow-up rate was expected until the T2 survey, and 166 participants were planned to be recruited.

### Statistical Analysis

The change in outcomes between the T1 and T2 surveys was calculated and compared by group using a *t* test. All participants who completed both the T1 and T2 surveys (complete cases) were included in the analysis according to their allocation. Sensitivity analyses were conducted to examine the robustness of the results and evaluate the variation in the assumptions of outliers, protocol compliance, missing values, outcome definition, clustering, contamination, and distribution of outcomes (Thabane et al., 2013).

Data from T1 to T3 in the intervention group were analyzed using a single-group, pre–post design. Due to missing values (loss to follow-up) and intraindividual correlations in the outcomes, a mixed model for repeated-measures was used in this analysis. Each time point was entered as a dummy variable (T1 vs T2 and T1 vs T3), and its fixed effects were estimated using the restricted maximum likelihood method. Degrees of freedom were adjusted using the Satterthwaite method. All analyses were performed using Stata 16 software (StataCorp LP, College Station, TX).

## Results

### Recruitment and Response

**Figure 1.**
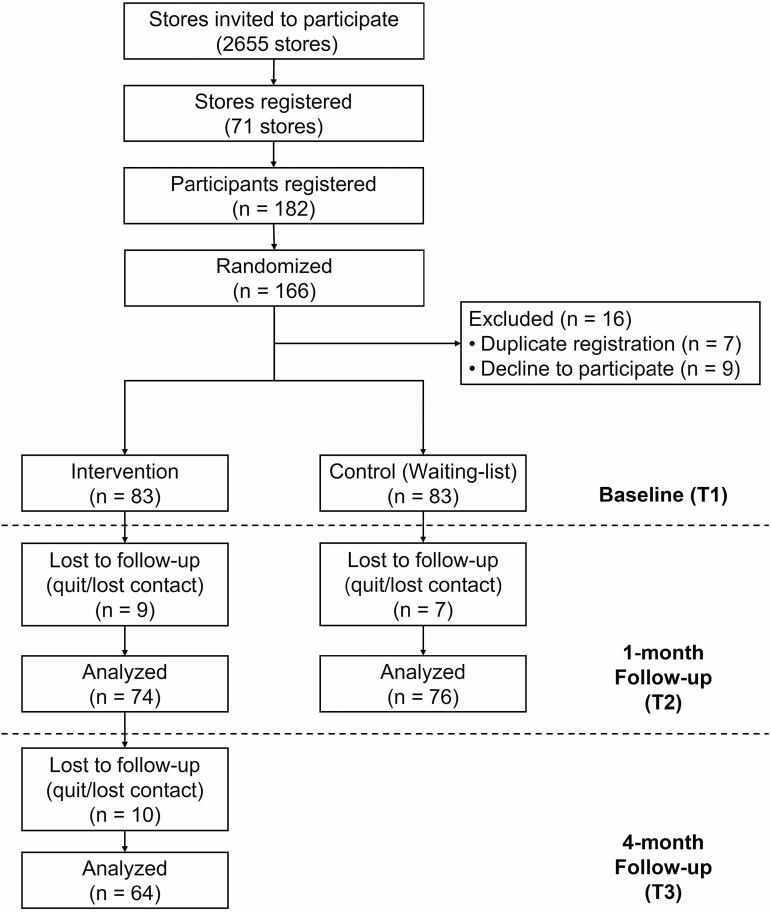
Consolidated Standards of Reporting Trials diagram.

The participant flow chart is shown in Figure 1. Direct mails were sent to 2,655 convenience stores, 71 (2.7%) of which registered. There were 166 (mean: 2.3 per store) participants randomized, and 150 participants completed the T2 survey. Ten participants were lost to follow-up in the T3 survey (intervention group only). No significant group differences were observed in the lost to follow-up rates across T1 to T2 surveys. Comparing complete cases with lost to follow-up cases, there were no significant baseline differences, except for helping behavior in the control group (Supplementary Table 2).

### Participant Characteristics

Table 1 shows the demographic variables and baseline outcomes for all the complete cases. Twenty-one participants (14.0%) were full-time employees, 101 (67.3%) were part-time employees, 16 (10.7%) were owners, and 12 (8.0%) were family members. Six participants (4.0%) had previously attended a dementia supporter training course, 45 (30.0%) had a family experience of dementia, and 12 (8.0%) had engaged in caregiving for family members with dementia. The average length of service at the current store was 3.3 years (standard deviation [*SD*]: 2.0), and the average age was 39.0 years (*SD*: 14.9). No significant differences were observed between the groups.

**Table 1.** Participant Characteristics (Analytical Sample, *N* = 150)

Variable	Intervention (*n* = 74)				Control (*n* = 76)					
	*n*	%	Mean	*SD*	*n*	%	Mean	*SD*	*p* ^a^	*p* ^b^
Gender										
Male	34	45.9			30	39.5			.722	
Female	39	52.7			45	59.2				
Educational background										
Junior high school	3	4.1			4	5.3			.635	
High school	35	47.3			31	40.8				
College/vocational school	16	21.6			18	23.7				
University or higher	20	27.0			19	25.0				
Employment										
Full-time	12	16.2			9	11.8			.531	
Part-time	46	62.2			55	72.4				
Owner	10	13.5			6	7.9				
Owner’s family	6	8.1			6	7.9				
Dementia supporter training										
Participated	71	95.9			73	96.1			.973	
Not participated	3	4.1			3	3.9				
Family dementia experience										
No	49	66.2			56	73.7			.549	
Yes, and cared	6	8.1			6	7.9				
Yes, but not cared	19	25.7			14	18.4				
Franchiser										
A	47	63.5			45	59.2			.118	
B	10	13.5			19	25.0				
C	8	10.8			9	11.8				
Others	9	12.2			3	3.9				
Age (year)			39.8	14.9			38.3	14.9		.531
Length of service (year)			3.3	2.0			3.3	2.1		.813
Working hours per week			28.0	21.1			25.4	17.2		.413
Baseline outcomes										
Attitude^c^			38.2	6.7			38.3	7.2		.898
Knowledge^d^			18.6	5.1			18.8	6.0		.809
Helping behavior^e^			2.45	0.88			2.53	0.75		.561

*Notes*: One case for age, two cases for gender, and two cases for educational background were missing. DKAS-J = Japanese-language version of the Dementia Knowledge Assessment Scale, *SD* = standard deviation.

^a^Chi-squared test.

^b^ *t* Test.

^c^Attitude toward people living with dementia: the range for possible score is 14–56.

^d^Knowledge of dementia (DKAS-J): the range for possible score is 0–36.

^e^Helping behavior for customers suspected of having dementia score: the range for possible score is 0–4.

### Process Evaluation

The results of the process evaluations are presented in Table 2. In the intervention group, 13 (15.7%) participants did not start the course, 3 (3.6%) partially completed the course, and 67 (80.7%) completed the ReDeSign course within 1 month. The median time for completion was 6 days, and the median screen time was 53 min. All the respondents to the response survey (*n* = 47) agreed to the item “(t)his course was useful,” and 97.9% of them agreed to “(y)ou were satisfied with the course overall.”

**Table 2.** Process Evaluation

Variable	*n*	%	Median	IQR
Progress (*n* = 83)^a^				
Not started	13	15.7		
Partly completed	3	3.6		
Completed	67	80.7		
Enrollment to completion (*n* = 67)^b^				
<1 day	16	23.9		
1–5 days	15	22.4		
6–10 days	9	13.4		
11–20 days	14	20.9		
21–31 days	13	19.4		
Screen time (*n* = 67)^b^				
31–60 min	46	68.7		
61–90 min	16	23.9		
91–120 min	2	3.0		
>120 min	3	4.5		
Enrollment to completion (days)^b^			6	16
Screen time (minutes)^b^			53	19
Useful (*n* = 47)^c^,^d^				
Strongly disagree	0	0.0		
Disagree	0	0.0		
Agree	14	29.8		
Strongly agree	33	70.2		
Satisfied (*n* = 47)^d^^,^^e^				
Strongly disagree	0	0.0		
Disagree	1	2.1		
Agree	17	36.2		
Strongly agree	29	61.7		

*Note*: IQR = interquartile range.

^a^Progress of the course among those allocated to the intervention group.

^b^The sample includes those who completed the course.

^c^“This course was useful to you.”

^d^The sample includes those who responded to the reaction survey after the course.

^e^“You were satisfied with the course as a whole.”

### Change in Outcomes by Group

The results of the main analysis are shown in Table 3 (*n* = 150). Compared to the control group, the intervention group showed a significantly greater change in the primary outcome, attitude toward people living with dementia (*p* < .001, Hedge’s *g* = 0.70 [95% confidential interval: 0.37–1.03]), and knowledge of dementia (*p* < .001, *g* = 0.59 [0.26–0.91]). There were no significant group differences for change in helping behavior (*p* = .120, *g* = 0.26 [−0.07 to 0.59]).

**Table 3.** Change From Baseline by Groups

Change in	Intervention (*n* = 74)		Control (*n* = 76)				Effect size		
	Mean	*SD*	Mean	*SD*	*p* ^a^		Hedge’s *g*	LLCI	ULCI
Attitude^b^	3.86	5.55	0.46	4.00	.000	***	0.70	0.37	1.03
Knowledge^c^	2.95	4.79	0.09	4.92	.000	***	0.59	0.26	0.91
Helping behavior^d^	0.28	0.78	0.06	0.89	.120		0.26	−0.07	0.59

*Notes*: DKAS-J = Japanese-language version of the Dementia Knowledge Assessment Scale, LLCI = lower limit 95% confidence interval, UCLI = upper limit 95% confidence interval, *SD* = standard deviation.

^a^ *t* Test.

^b^Attitude toward people living with dementia.

^c^Knowledge of dementia (DKAS-J).

^d^Helping behavior for customers suspected of having dementia score.

***p < .001.

### Sensitivity Analysis

All sensitivity analyses found qualitatively equivalent results in the change of attitudes toward the main analysis (Supplementary Figures 1–6). Subgroup analyses for attitude change generally showed similar results to the main analysis, although they suggested that the effect may be smaller when employment status was owner.

For helping behavior, significant group differences were observed in the analysis, excluding cases where contamination could occur (i.e., those who were allocated to the control group and whose store had intervention group participants [*n* = 52]). For the other assumptions, the results were qualitatively equivalent to those of the main analysis.

### Trend in Intervention Group

Table 4 shows the trends in the outcomes of the intervention group. The single-group repeated-measures model found that all outcomes improved significantly from T1 to T2 (*p* < .001). However, there was a slight decline from T2 to T3, attitudes (*p* < .001), knowledge (*p* = .003), and helping behaviors (*p* = .008) showed significant improvement from T1 to T3.

**Table 4.** Outcome Measures Across Time Points in the Intervention Group

Measure	Time	*n*	Mean	*SD*	Coefficient^a^	LLCI	ULCI	*p*	
Attitude^b^	T1	83	38.10	6.70	REF				
	T2	73	42.10	6.91	3.89	2.74	5.04	.000	***
	T3	66	41.40	6.70	3.02	1.83	4.21	.000	***
Knowledge^c^	T1	83	18.71	5.02	REF				
	T2	73	21.42	5.98	2.83	1.66	4.01	.000	***
	T3	66	20.35	6.20	1.87	0.65	3.09	.003	**
Helping behavior^d^	T1	81	2.46	0.89	REF				
	T2	68	2.77	0.81	0.30	0.13	0.47	.001	***
	T3	62	2.73	0.86	0.25	0.07	0.43	.008	**

*Notes*: LLCI/ULCI indicates 95% lower/upper limit confidence interval. DKAS-J = Japanese-language version of the Dementia Knowledge Assessment Scale, *SD* = standard deviation.

^a^Mixed-model for repeated-measures, where T1 score is the reference level.

^b^Attitude toward people living with dementia.

^c^Knowledge of dementia (DKAS-J).

^d^Helping behavior for customers suspected of having dementia score.

***p < .001; **p < .01.

## Discussion

The present study found that a dementia-friendly training program using a mobile microlearning approach for convenience store workers significantly improved attitudes toward people living with dementia and their knowledge of dementia. In addition, a significant increase in helping behavior was observed after the intervention; however, it could not be concluded that this gain was caused by the intervention.

The main results of the significant improvement in knowledge and attitude are consistent with those of several existing pre–post or qualitative studies (Matsumoto et al., 2021). Sensitivity analysis confirmed the robustness of the results. However, the only previous RCT (i.e., Kim et al., 2021) reported no significant intervention effects. The major reason for the difference in the results between the present study and that of Kim et al. (2021) could be selection bias. A smaller proportion of the participants in our study had a family history of dementia or caregiving experience than those in the aforementioned study, and the participants’ baseline attitude scores were similar to those in previous studies of community residents (Igarashi et al., 2020; Kuroda et al., 2011). In addition, the loss to follow-up rate in our study was lower, and there was no significant association between attitude and loss to follow-up rate. The participants analyzed in this study were not biased in their baseline stigma levels, and thus, the intervention effects were more likely to be demonstrated.

The effect size on the primary outcome was 0.70 for attitude and 0.59 for knowledge, interpreted as medium (Brydges, 2019). Previous studies on dementia-friendly training programs using the same scale reported smaller effect sizes (Igarashi et al., 2020; Sari et al., 2020). ReDeSign was hypothesized to achieve greater stigma reduction because it is a theory-based program that integrates education consisting of lectures, and quizzes, and contact consisting of videos and simulations. The combination of education and contact has been identified as the most effective way to reduce stigma regarding mental illness (Griffiths et al., 2014; Kim et al., 2021). Microlearning benefits memory retention (Göschlberger & Bruck, 2017; Jahnke et al., 2020), and documentary videos, along with the simulation game, may have contributed to the improvement of attitudes (Janoušková et al., 2017; Miles & Crisp, 2014; Yamaguchi et al., 2013). This combination of content should continue to be used for dementia-friendly training in the future.

One of the new findings of this study is that helping behavior increased after the intervention. The program design based on the bystander intervention model and measurement of behavior are strengths of this study. The group difference was not significant because of the slight increase in the control group, which suggested that the sensitivity analysis was caused by contamination. For example, if the store manager was allocated to the intervention group, they might have instructed their staff in the control group to help or report on customers with dementia. While individual-level randomization was adopted in this study to facilitate recruitment, future evaluations could adopt a cluster-randomized design to improve the estimation accuracy of the intervention effects on helping behavior. In the future dissemination phase, as the training program will be introduced organizationally, the intervention would resemble cluster randomization. Store-level engagement might enhance the intervention effect through collaborative learning, organizational awareness, and goal setting.

This study contributes to the development of the model for promoting helping behavior, though the lack of a significant increase in helping behavior did not allow us to test the program theory. The small increase in helping behavior suggests the need for other approaches, including intervention on responsibility. Our program did not include any content that affected the participants’ feeling of responsibility (the third step in the bystander intervention model), assuming that store workers are more likely to feel responsible for helping than public bystanders. In Japan, more than half of local governments have concluded agreements with companies to share their common goal of supporting older people and stipulates store staff activities that support older adults directly or indirectly (Nakamura et al., 2018). These agreements encourage—though not mandating—helping behavior by the companies. Because store workers may not be aware of these agreements, educating them on such agreements is one possible approach that could influence their feelings of responsibility. The model of promoting helping behavior is developing and will benefit from additional investigation through interviews and ethnographic observations with store workers.

There are several limitations to this study other than the possibility of contamination. First, the representativeness of the participants was restricted, even though it is unlikely that the stigma levels were biased. The participation rate at the store level was 2.7%. Based on the fact that the average number of workers in a convenience store is 15.6 (Ministry of Internal Affairs and Communications, 2016), the participation rate in the store was estimated to be about 15%. One of the reasons for the low participation rate was that the study was conducted during the COVID-19 epidemic period, and the recruitment method was limited to direct mail. Second, because the trial was not blinded and all outcomes were self-reported, it is possible that the effectiveness of the ReDeSign course was not accurately measured. Participants in the intervention group may have been more susceptible to social desirability bias regarding their attitudes and helping behaviors after the intervention. It is recommended that future studies use implicit attitude measurements for attitudes (Kane et al., 2020) and video recording or direct observation for helping behavior to reduce the social desirability bias. Third, because the contents were packaged and sequentially interrelated, it was not possible to evaluate the individual contents. Evaluation through factorial experiments can contribute to refining the program and improving its efficiency (Collins et al., 2014).

Despite these limitations, to the best of our knowledge, the present study is the first RCT to demonstrate the effectiveness of dementia-friendly training for the general public. This is also the first study to demonstrate an increase in helping behaviors. Improved knowledge and attitudes are beneficial not only in their role as store workers but also in their help-seeking when their family members or they experience dementia (Matsumoto et al., 2021).

Adopting a mobile microlearning approach, ReDeSign has a relative advantage over existing dementia-friendly training in terms of time constraints, and thus, dissemination. Dementia-friendly training for employees is often implemented as a corporate social responsibility, but it is also expected to increase a company’s competitive advantage and revenue (Alzheimer’s Society, 2017). Once ReDeSign is available to the public on the Internet, it will be a promising option for retailers, such as convenience stores, to introduce dementia-friendly training. Customization to adapt to existing in-house training systems and workplace contexts is desirable.

While this study focused only on convenience stores, similar programs could be applied to other general occupations in which workers interact with people living with dementia. Dementia-friendliness is not always a priority for general occupations, which include supermarkets, transportation, and banks. Additionally, dementia-friendly training always faces time constraints. Effective dementia-friendly training, when disseminated in the community, would help enable people living with dementia to maintain social participation.

## Supplementary Material

gnac182_suppl_Supplementary_Material
