# Supplementary material for: A Randomized Control Trial for ReDeSign: A Dementia-Friendly Mobile Microlearning Training for Store Workers in Japan
Source: Gerontologist. 2022 Dec 12;63(8):1300–10. doi: 10.1093/geront/gnac182 (PMC10474589; doi:10.1093/geront/gnac182)
Supplement: gnac182_suppl_Supplementary_Material [file gnac182_suppl_supplementary_material.docx]

| **Supplementary Table 1. Helping behavior items and distribution** | | | | | | | | | | | | |
| --- | --- | --- | --- | --- | --- | --- | --- | --- | --- | --- | --- | --- |
|  | | Encounter within the past month (N=150) | | | | | | | | | | |
|  |  | Not encountered | |  | Encountered | | | | | | | |
|  |  | n | % |  | n | % |  | Helping behavior ^a^ | | | | |
|  |  |  |  |  |  |  |  | Never (1) | Sometimes (2) | Often (3) | Always (4) | Mean |
|  |  |  |  |  |  |  |  | n | n | n | n |  |
| *A customer who ^b^* | |  |  |  |  |  |  |  |  |  |  |  |
|  | has lost his/her way home | 134 | 89.3 |  | 16 | 10.7 |  | 0 | 6 | 2 | 8 | 3.13 |
|  | purchases the same items repeatedly | 91 | 60.7 |  | 59 | 35.5 |  | 21 | 9 | 14 | 15 | 2.39 |
|  | talks about something that has nothing to do with the store | 51 | 34.0 |  | 99 | 59.6 |  | 19 | 26 | 29 | 25 | 2.61 |
|  | takes a long time to pay | 10 | 6.7 |  | 140 | 84.3 |  | 29 | 34 | 32 | 45 | 2.66 |
|  | gives bills even though he/she has coins | 26 | 17.3 |  | 124 | 74.7 |  | 57 | 31 | 21 | 15 | 1.95 |
|  | cannot hold a conversation well | 69 | 46.0 |  | 81 | 48.8 |  | 19 | 35 | 11 | 16 | 2.30 |
|  | tells the same story repeatedly | 78 | 52.0 |  | 72 | 43.4 |  | 20 | 22 | 18 | 12 | 2.31 |
|  | asks where the item is before looking for it him/herself | 17 | 11.3 |  | 133 | 80.1 |  | 9 | 26 | 21 | 77 | 3.25 |
|  | were suspected of having dementia | 79 | 52.7 |  | 71 | 42.8 |  | 12 | 27 | 15 | 17 | 2.52 |
|  |  |  |  |  | n | % |  |  |  |  |  |  |
| Encountered any of the situations above | |  |  |  | 147 | 88.6 |  |  |  |  |  |  |
| Offer helping behavior on any of the situations above ^c^ | | | | | 140 | 84.3 |  |  |  |  |  |  |
|  |  |  |  |  | Mean | SD |  |  |  |  |  |  |
| The number of encountered situations | |  |  |  | 5.30 | 2.17 |  |  |  |  |  |  |
| Helping behavior score ^d^ | |  |  |  | 2.49 | 0.81 |  |  |  |  |  |  |
| a) The definition of helping behavior includes direct helping behavior and information sharing with supervisors, coworkers, and administrative agencies, b) These items were collected through an open-ended survey of convenience store managers (n=196) who experienced difficulties in responding to the older customers. Through subsequent preliminary research, the list was constructed by excluding items that were unlikely to be attributable to dementia, were encountered infrequently, or had low internal consistency and criterion-related validity, c) Those who encountered and offered helping behavior sometimes, often, or always, d) Average score of helping behavior (1-4) for 9 items. "Not encountered" items were treated as missing values. | | | | | | | | | | | | |

| **Supplementary Table 2. Complete case and lost to follow-up by arms** | | | | | | | | | | | | | | |
| --- | --- | --- | --- | --- | --- | --- | --- | --- | --- | --- | --- | --- | --- | --- |
|  | | Intervention | | | | |  | Control | | | | |  | |
|  |  | Complete case (n=74) | | Lost to  follow-up (n=9) | |  |  | Complete case  (n=76) | | Lost to  follow-up (n=7) | |  |  | |
|  |  | n | % | n | % | *p^a^* |  | n | % | n | % | *p^a^* |  | |
| *Gender* | |  |  |  |  |  |  |  |  |  |  |  |  | |
|  | Male | 34 | 45.9 | 4 | 44.4 | 0.933 |  | 30 | 39.5 | 5 | 71.4 | 0.258 |  | |
|  | Female | 39 | 52.7 | 5 | 55.6 |  |  | 45 | 59.2 | 2 | 28.6 |  |  | |
| *Educational background* | | | |  |  |  |  |  |  |  |  |  |  | |
|  | Junior high school | 3 | 4.1 | 1 | 11.1 | 0.807 |  | 4 | 5.3 | 0 | 0.0 | 0.949 |  | |
|  | High school | 35 | 47.3 | 5 | 55.6 |  |  | 31 | 40.8 | 4 | 57.1 |  |  | |
|  | College/Vocational school | 16 | 21.6 | 1 | 11.1 |  |  | 18 | 23.7 | 2 | 28.6 |  |  | |
|  | University or higher | 20 | 27.0 | 2 | 22.2 |  |  | 19 | 25.0 | 1 | 14.3 |  |  | |
| *Employment* | |  |  |  |  |  |  |  |  |  |  |  |  | |
|  | Full-time | 12 | 16.2 | 1 | 11.1 | 0.452 |  | 9 | 11.8 | 3 | 42.9 | 0.115 |  | |
|  | Part-time | 46 | 62.2 | 4 | 44.4 |  |  | 55 | 72.4 | 3 | 42.9 |  |  | |
|  | Owner | 10 | 13.5 | 2 | 22.2 |  |  | 6 | 7.9 | 1 | 14.3 |  |  | |
|  | Owner's family | 6 | 8.1 | 2 | 22.2 |  |  | 6 | 7.9 | 0 | 0.0 |  |  | |
| *Dementia Supporter training* | | | | |  |  |  |  |  |  |  |  |  | |
|  | Participated | 71 | 95.9 | 9 | 100.0 | 0.538 |  | 73 | 96.1 | 6 | 85.7 | 0.222 |  | |
|  | Not participated | 3 | 4.1 | 0 | 0.0 |  |  | 3 | 3.9 | 1 | 14.3 |  |  | |
| *Family dementia experience* | | | | |  |  |  |  |  |  |  |  |  | |
|  | No | 49 | 66.2 | 5 | 55.6 | 0.062 |  | 56 | 73.7 | 4 | 57.1 | 0.639 |  | |
|  | Yes, and cared | 6 | 8.1 | 3 | 33.3 |  |  | 6 | 7.9 | 1 | 14.3 |  |  | |
|  | Yes, but not cared | 19 | 25.7 | 1 | 11.1 |  |  | 14 | 18.4 | 2 | 28.6 |  |  | |
| *Franchiser* | |  |  |  |  |  |  |  |  |  |  |  |  | |
|  | A | 47 | 63.5 | 8 | 88.9 | 0.378 |  | 45 | 59.2 | 2 | 28.6 | 0.276 |  | |
|  | B | 10 | 13.5 | 0 | 0.0 |  |  | 19 | 25.0 | 2 | 28.6 |  |  | |
|  | C | 8 | 10.8 | 1 | 11.1 |  |  | 9 | 11.8 | 2 | 28.6 |  |  | |
|  | Others | 9 | 12.2 | 0 | 0.0 |  |  | 3 | 3.9 | 1 | 14.3 |  |  | |
|  | | Mean | SD | Mean | SD | *p^b^* |  | Mean | SD | Mean | SD | *p^b^* |  | |
| Age (year) | | 39.8 | 14.9 | 38.9 | 17.2 | 0.865 |  | 38.3 | 14.9 | 37.9 | 16.0 | 0.945 |  | |
| Length of service (year) | | 3.258 | 1.985 | 2.375 | 1.985 | 0.211 |  | 3.337 | 2.073 | 3.643 | 1.773 | 0.707 |  | |
| Working hours per week | | 28.007 | 21.104 | 29.167 | 21.36 | 0.877 |  | 25.428 | 17.236 | 33.214 | 11.701 | 0.247 |  | |
| *Baseline Outcomes* | | |  |  |  |  |  |  |  |  |  |  |  | |
|  | Attitude ^c^ | 38.2 | 6.7 | 37.7 | 7.0 | 0.837 |  | 38.3 | 7.2 | 42.4 | 4.7 | 0.141 |  | |
|  | Knowledge ^d^ | 18.6 | 5.1 | 19.4 | 4.5 | 0.645 |  | 18.8 | 6.0 | 21.3 | 6.3 | 0.306 |  | |
|  | Helping behavior ^e^ | 2.45 | 0.88 | 2.51 | 0.98 | 0.85 |  | 2.53 | 0.75 | 3.11 | 0.43 | 0.046 | * | |
| Note. Column percentages are shown. One case for age, two cases for gender, and two cases for educational background were missing. * p<0.05 a) Chi-squared test, b) t-test, c) Attitude toward people living with dementia: The range for possible score is 14-56, d) Knowledge of dementia (DKAS-J): The range for possible score is 0-36, e) Helping behavior for customers suspected of having dementia score: The range for possible score is 0-4. | | | | | | | | | | | | | |  |

## Supplementary Figures

Sensitivity analysis

##### Subgroup Analysis

Subgroup analyses were conducted based on gender (male, female), age (10-20s, 30-40s, 50-70s), employment status (full-time, part-time, owner, owner family), baseline attitudes toward people living with dementia, knowledge of dementia, helping behavior toward customers suspected of having dementia, population density around the store, and course completion time.

Continuous variables were divided by the grand mean (low, high).

The aim of the subgroup analysis based on course completion time was to examine the impact of the time between course completion and T2 survey. The intervention group was divided into early (more than three weeks from course completion to T2 survey) or late learners (less than three weeks), while the control group was not divided into groups and analyzed in the same way as the main analysis.

The aim of the subgroup analysis based on population density was to examine the impact of the community context. The population density of the municipality in which the stores were located was obtained from the government statistics. The results are shown in Supplementary Figures 1, 2, and 3, respectively. Owing to the small sample size, the power was insufficient. Based on the point estimates, we interpreted the results as qualitatively equivalent to the main analysis.

##### Sensitivity analysis

In this study, we conducted a sensitivity analysis of the scenarios presented in the following section based on Thabane et al. (2013). The results are shown in Supplementary Figures 4, 5, and 6, respectively. The regression coefficients and 95% confidence intervals estimated for each analysis are shown as forest plots. All independent variables in the regression analysis were dummy variables, with intervention group = 1 and control group = 0. The results of the primary analysis, being t-test in the manuscript, were shown as regression coefficient of “complete cases,” because of comparability with the other results.

##### Outlier

We conducted regressions excluding cases with outcome outliers (change scores). The interquartile range (IQR) methods identified values less than the 25th percentile -1.5 IQR or greater than the 75th percentile +1.5 IQR as outliers for each outcome.

##### Protocol compliance

Nine participants (12.2 %) in the intervention group (n=74) responded to the T2 survey without completing the intervention. None of the participants in the control group received the intervention before the T2 survey was conducted. The “per-protocol” analysis excluded cases that were assigned to the intervention group but did not receive the intervention (n=9). “As treated” analysis treated the same cases as in the control group.

##### Missing data

Because this study used a web-based survey, missing data cannot occur within a single case at a single time point. There were three types of missing data in this study: (1) failure to follow-up, (2) helping behavior items when not encountering the situation, and (3) "unwilling to answer" responses for age, gender, and educational background.

##### Lost to follow-up

There were 16 cases (9.6%) of missing data due to (1) loss to follow-up from T1 to T2.

##### Complete case analysis (Main analysis)

A complete case analysis was used for the main analysis. A complete case analysis provides unbiased estimates under the assumption that missing values occur completely at random (MCAR). The absence of variables (except for one variable in the control group) was significantly associated with loss to follow-up, supporting the MCAR assumption.

##### Best-worst and Worst-best methods

The best-worst method generates the "best-case scenario" by substituting a beneficial outcome for the missing data in the intervention group and an adverse outcome for the missing data in the control group. Conversely, the worst method created a "worst-case scenario" by substituting an adverse outcome for the intervention group and a beneficial outcome for the control group.

In this study, mean + 1 SD was substituted as a beneficial outcome and mean − 1 SD as an adverse outcome (when a higher score is beneficial) for the outcomes of continuous variables. We interpreted the results as qualitatively equivalent to the main analysis when the point estimates from the best and worst methods were within the 95% confidence interval of the main analysis.

##### Multiple Imputation

Because only a single outcome value was missing in each analysis and the normal distribution of outcomes was assumed, this analysis used linear regression as the assignment algorithm. We assumed that the reasons for missing data (i.e., not responding to T2) were (1) a lack of interest in dementia-friendly training, (2) being busy at work, (3) unfamiliarity with smartphones/PCs, and (4) franchisor characteristics. As auxiliary variables corresponding to these reasons, we used the following: (1) attitude toward PLWD, knowledge of dementia, experience of attending a dementia supporter training course, and family experience of dementia at baseline; (2) working hours (per week) and employment status at baseline; (3) age and educational background at baseline; and (4) franchisors (A, B, C, others). Sex, allocation group, and baseline values for each outcome were also included as auxiliary variables. The STATA "mi command" was used for the analysis, and 20 imputation datasets were generated for each dataset.

##### Missing values of helping behavior due to non-encountering situations

If a participant did not encounter a situation, the helping behavior score for that item was missing. The main analysis excluded missing data and used the mean of the remaining items as the scale score (single-mean imputation). Because a high proportion of these items are missing (non-encountered), it is not suitable for estimation using complete case analysis or other imputation methods.

Instead, we treated the ninth item, "a customer who was suspected of having dementia," as a global item, and conducted a complete case analysis using the change score of this item. The ninth item was missing at T1 in 86/166 (51.8%) cases and at T2 in 100/140 (69.4%) cases, and both T1 and T2 were complete in 44/140 (31.4%) cases. The result is shown in Figure C3 as “global item.”

##### Missing values for age, gender, and educational background due to "do not want to answer."

Sensitivity analysis was not conducted because the number of missing values was small in all cases (1, 2 and 2, respectively).

##### Definition of outcomes

The alternative definition of the outcomes, other than a change from T1 to T2, is the value at T2. Analysis of covariance (ANCOVA) was conducted, with group allocation as the independent variable, the value at T1 as the covariate, and the value at T2 as the dependent variable. Although no imbalance between the groups was observed at baseline in this study, estimation by ANCOVA also assessed the impact of imbalance.

In addition, we conducted mixed-effects models for repeated measures (MMRM) that nested T1 and T2 data to the individuals. This can also be interpreted as a sensitivity analysis of missing data due to loss to follow-up at T2 under the missing-at-random assumption.

##### Clustering

Multilevel analysis was conducted, adjusting for stores (clusters) as a random effect. The fixed effects of the allocation variable were estimated using the restricted maximum-likelihood method. Degrees of freedom were adjusted using the Satterthwaite method.

##### Contamination

Participants in the control group (n=76) were classified into two categories: (1) those whose stores had only control group participants (n=24) and (2) those whose stores had intervention group participants (n=52). "Impossible contamination" analysis included only the former category as the control group, and "possible contamination" analysis included only the latter. The intervention group and statistical analyses were the same as in the main analysis.

Contamination leads to an attenuation of the effect of the intervention. The presence of contamination was supported when the estimated intervention effect in the impossible contamination analysis was greater than that in the "possible contamination" analysis.

##### Assumption of distribution

The t-test and linear regression analysis assumed a normal distribution of variables. To evaluate the variability of this assumption, we tested the group differences for each outcome using the Wilcoxon rank-sum test, which does not assume a normal distribution. The results did not differ from the main analysis: p < 0.001 for attitude, p < 0.001 for knowledge, and p = 0.151 for helping behavior.

Supplementary
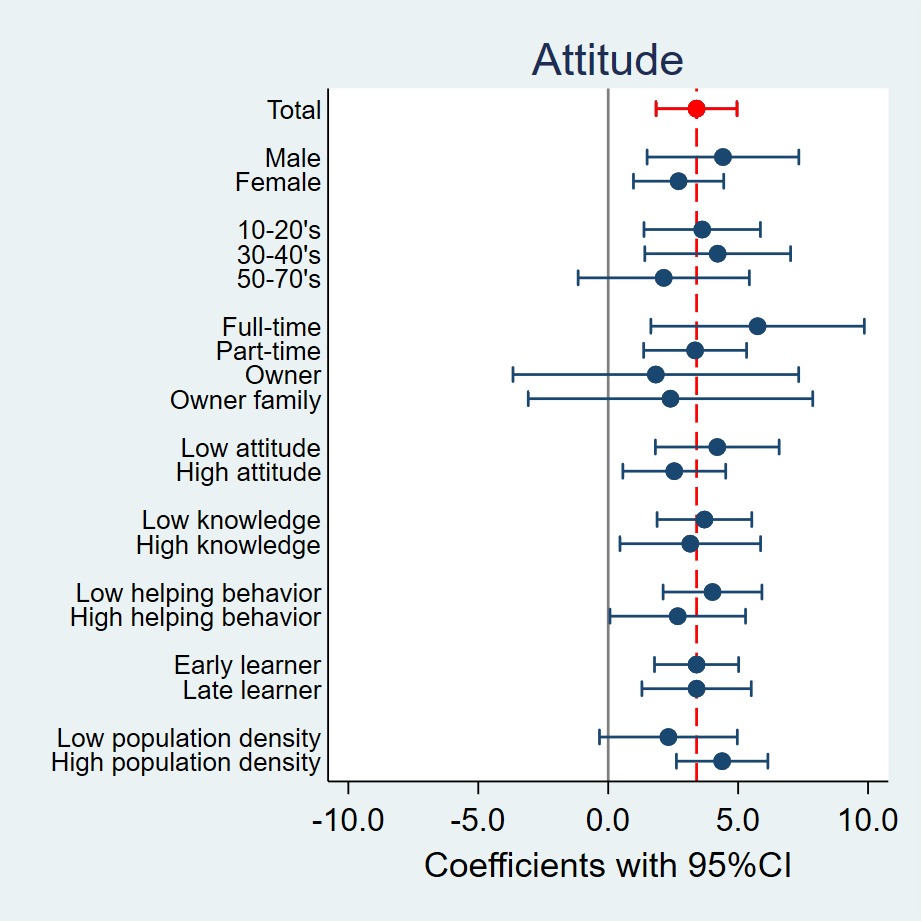
Figure 1. Subgroup analysis for the change of attitude toward people living with dementia


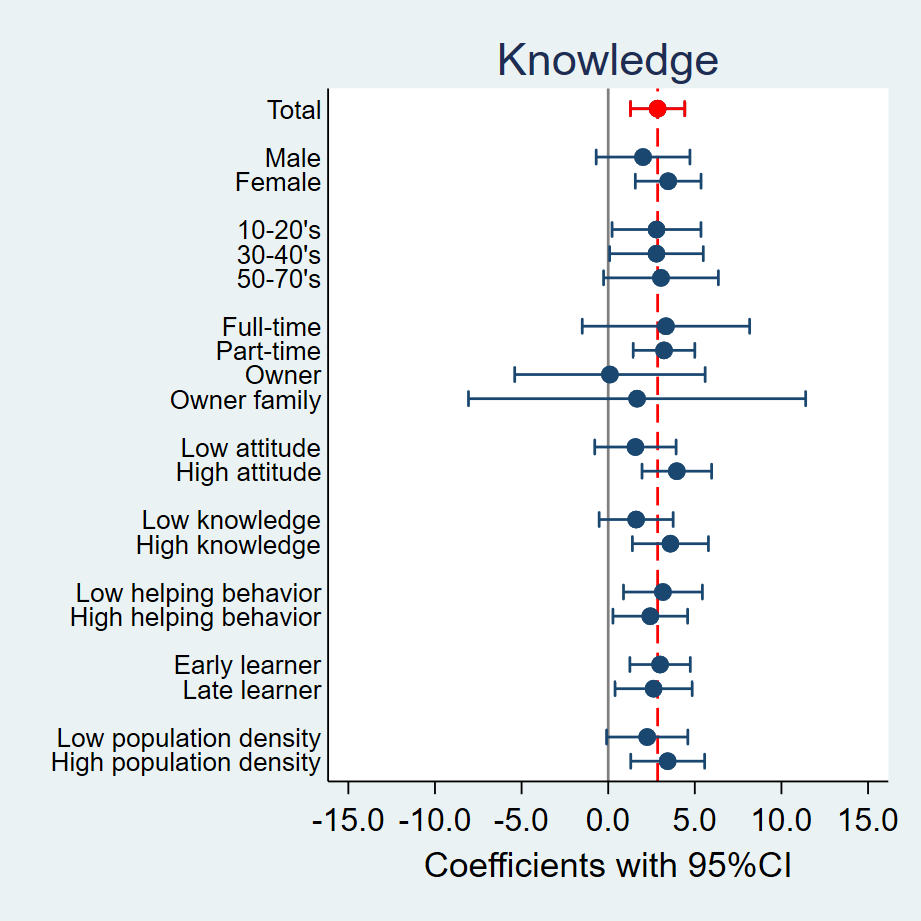


Supplementary Figure 2. Subgroup analysis for the change of knowledge of dementia


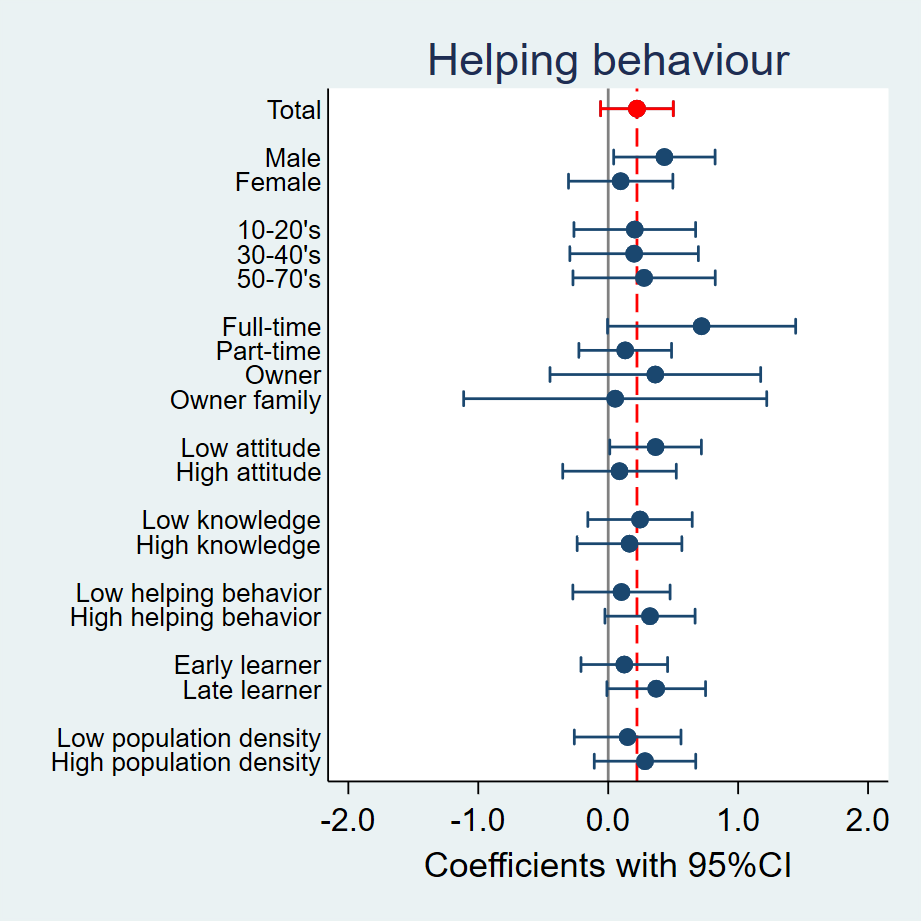


Supplementary Figures 3. Subgroup analysis for the change of helping behavior


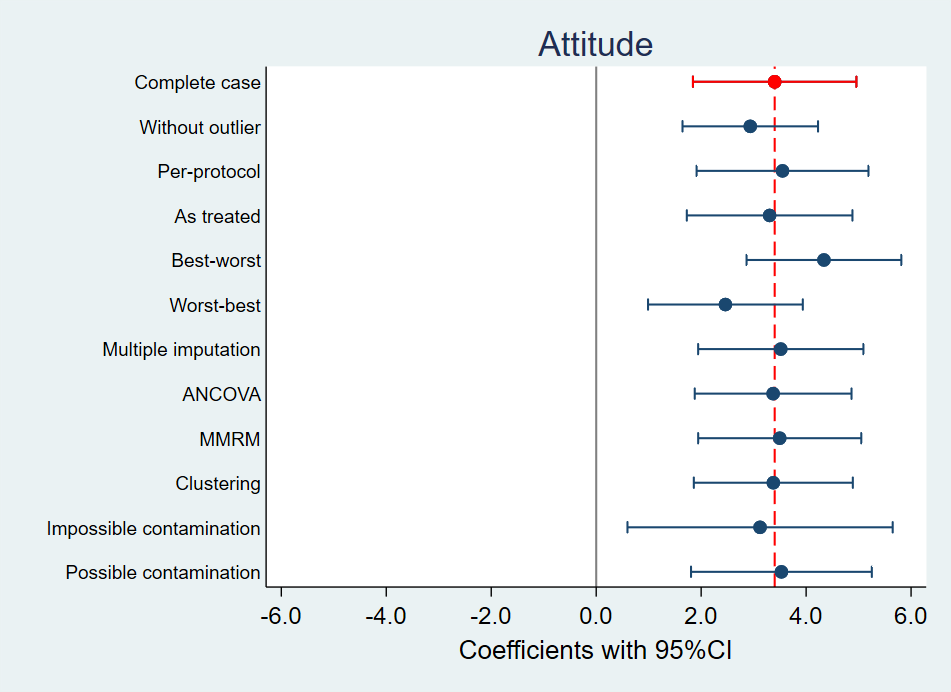
Supplementary Figure 4. Sensitivity analysis for the change of attitude toward people living with dementia


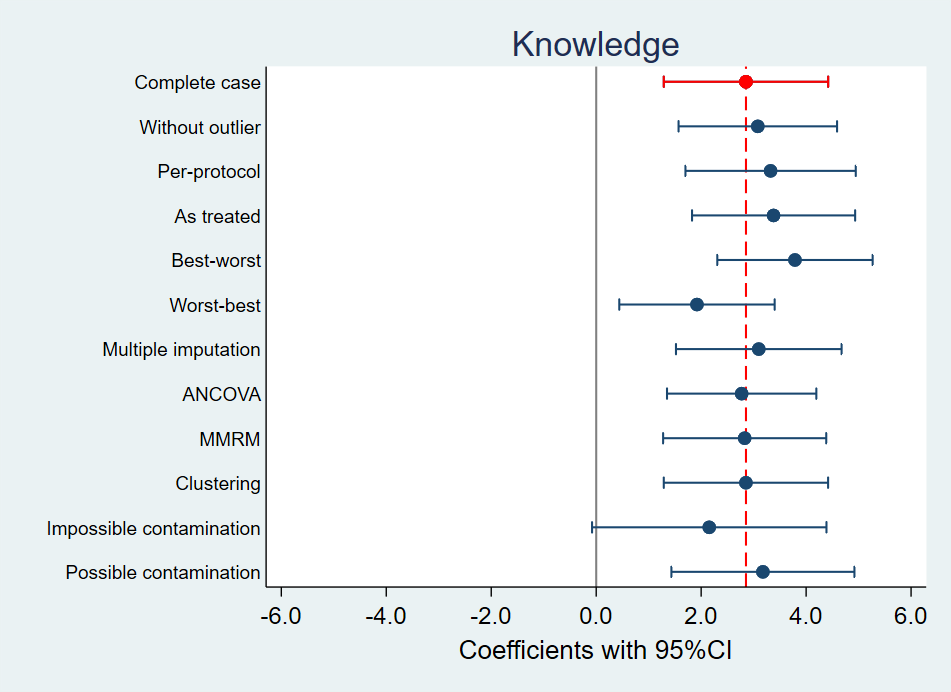


Supplementary Figure 5. Sensitivity analysis for the change of knowledge of dementia


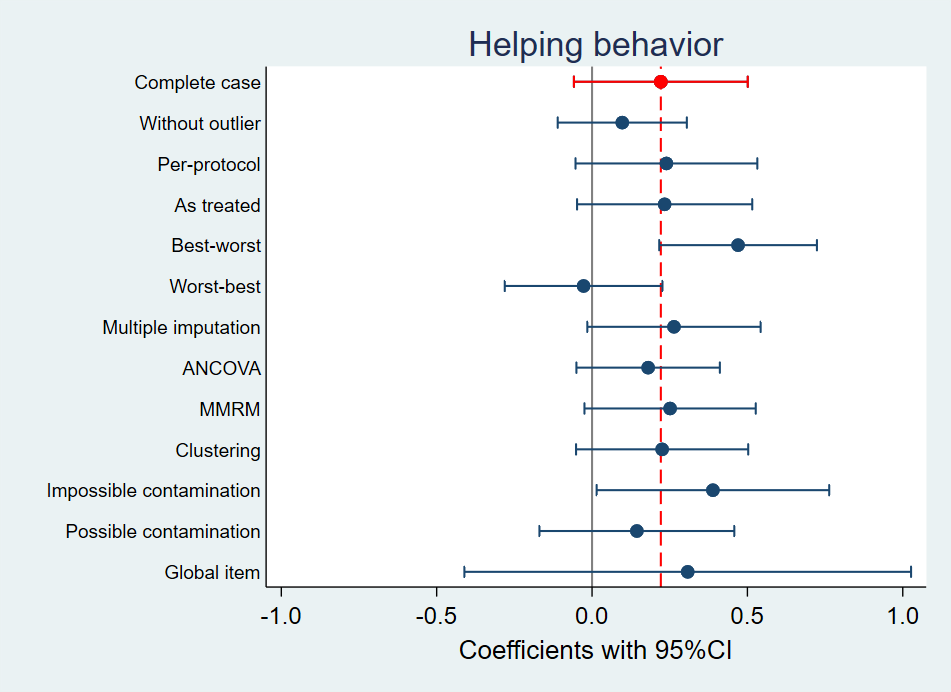
Supplementary Figure 6. Sensitivity analysis for the change of helping behavior
